# Statins for progression of aortic valve stenosis and the best evidence for making decisions in health care

**DOI:** 10.1590/S1516-31802011000100008

**Published:** 2011-01-06

**Authors:** Luciana Thiago, Selma Rumiko Tsuj, Álvaro Nagib Atallah, Maria Eduarda dos Santos Puga, Aécio Flávio Teixeira de Góis

**Affiliations:** I MD. Cardiologist. Assistant lecturer, Faculdade de Medicina de Marília (Famema), Marília, São Paulo, and member of the Research Group, Brazilian Cochrane Center, São Paulo, Brazil.; II MD, PhD. Psychiatrist. Professor, Faculdade de Medicina de Marília (Famema), Marília, São Paulo, and member of the Research Group, Brazilian Cochrane Center, São Paulo, Brazil.; III MD. Nephrologist. Titular professor of Evidence-Based Medicine and Emergency Medicine, Universidade Federal de São Paulo – Escola Paulista de Medicina (Unifesp-EPM), São Paulo, and director of the Brazilian Cochrane Center, São Paulo, Brazil.; IV MSc. Librarian. Research assistant, Brazilian Cochrane Center, São Paulo, Brazil.; V MD, PhD. Cardiologist. Head of Department of Emergency Services and Emergency Intensive Care Unit, Hospital São Paulo, Universidade Federal de São Paulo – Escola Paulista de Medicina (Unifesp-EPM), São Paulo, and member of the Research Group, Brazilian Cochrane Center, São Paulo, Brazil.

**Keywords:** Aortic valve stenosis, Hydroxymethylglutaryl-CoA reductase inhibitors, Atherosclerosis, Disease progression, Review [publication type], Estenose da valva aórtica, Inibidores de hidroximetilglutaril-CoA redutases, Aterosclerose, Avanço da doença, Revisão

## Abstract

In the Western world, calcified aortic valve stenosis is the most common form of valvular heart disease, affecting up to 3% of adults over the age of 75 years. It is a gradually progressive disease, characterized by a long asymptomatic phase that may last for several decades, followed by a short symptomatic phase associated with severe restriction of the valve orifice. Investigations on treatments for aortic valve stenosis are still in progress. Thus, it is believed that calcification of aortic valve stenosis is similar to the process of atherosclerosis that occurs in coronary artery disease. Recent studies have suggested that cholesterol lowering through the use of statins may have a salutary effect on the progression of aortic valve stenosis.

## RESUMO

No mundo ocidental, a estenose da valva aórtica calcificada é a forma mais comum de doença cardíaca valvar, afetando até 3% dos adultos com idade acima de 75 anos. É uma doença gradual e progressiva, caracterizada por uma longa fase assintomática, podendo durar várias décadas, seguida de uma curta fase sintomática associada à restrição grave do orifício da valvar. A investigação sobre o tratamento da estenose da valva aórtica ainda está em curso. Desta forma, acredita-se que a calcificação da estenose da valva aórtica seja semelhante ao processo de aterosclerose que ocorre na doença arterial coronariana. Estudos recentes têm sugerido que a redução do colesterol pelo do uso das estatinas pode ter um efeito salutar sobre a progressão da estenose da valva aórtica.

## INTRODUCTION

Aortic valve stenosis in adults is characterized by degenerative changes to the valve cusps that hinder proper emptying of the left ventricle and promote the development of ventricular hypertrophy. The main causes of valve disease are congenital, rheumatic and degenerative (i.e. age-related). Currently, the main types of aortic valve stenosis are bicuspid and tricuspid calcification.^1^

Aortic calcification often presents greater development in patients with bicuspid aortic valves, a condition that affects 1-2% of the population and predominates in males.^1^ When this situation develops, it happens relatively early, at the age of 50-60 years.^1^ However, the most frequent form of presentation, tricuspid calcified aortic stenosis, occurs in 2-7% of adults over 65 years of age.^1^ In the latter patients, as the obstruction gradually develops, the left ventricle adapts to pressure overload through a hypertrophy process that results in increased wall thickness, while the chamber volume is maintained. The development of concentric hypertrophy appears to be an appropriate and beneficial adaptation to compensate for high intracavitary pressures. However, if this process is insufficient, the ejection capacity becomes reduced and left ventricular systolic dysfunction occurs. As a consequence of ventricular hypertrophy and decreased compliance of the chamber, there is also an increase in diastolic pressure without dilatation of the chamber. Thus, this increased pressure is also a reflection of the left ventricular diastolic dysfunction.^1^

This situation of a hypertrophied heart may diminish the coronary blood flow per gram of muscle and also exposes the limited coronary reserve. Thus, under conditions of exercise or tachycardia, maldistribution of blood flow and coronary subendocardial ischemia may occur. Concomitantly with this process, myocardial fibrosis is another early morphological alteration in patients with aortic valve stenosis, and this provides substrates for arrhythmias and gives rise to progression of heart failure and sudden death.^1^

The historical concept of aortic valve calcification was that it was a passive process, with serum calcium associated with the surface of the valve leaflet, thus forming a nodule. However, epidemiological studies, histopathological studies and recent experimental evidence have indicated that calcification of the aortic valve is an active biological process rather than a passive process within the leaflet, thus causing regulated bone formation.^2^

Similarities in risk factors strongly suggest that there are similarities in the underlying process of development and progression between calcific aortic valve stenosis and coronary atherosclerosis. The risk factors shared by these two disorders include hypertension, dyslipidemia (low levels of high-density lipoprotein; LDL), male gender, smoking and diabetes mellitus. Important histopathological evidence that calcific aortic valve stenosis develops in the same way as seen in atherosclerosis was reported by O'Brien et al.,^3^ who found that apolipoproteins B and E were present in early lesions and advanced aortic valve stenosis, but not in the region of normal valves, and found the same pattern of lipoproteins in coronary atherosclerosis.^2,3^

In the Western world, calcified aortic valve stenosis is the most common form of valvular heart disease, affecting 3-5% of adults over 75 years of age. It is a gradually progressive disease, characterized by a long asymptomatic phase lasting several decades with a risk of death of less than 1% per year, followed by a shorter symptomatic phase that is associated with severe restriction of the valve orifice. The progression rate varies, but the average reduction in the valve orifice is 0.1 cm^2^ each year.^4^

In degenerative calcific aortic valve stenosis, the progression begins at the base of the leaflets and heads towards the hole. All three cusps are usually affected, but one may be more dominant. In calcification, early progression starts in the middle raphe. In rheumatic disease, progression is greatest in the commissures. Significant calcification is associated with relatively rapid progression.^4^

The clinical criteria involved in diagnosing calcified aortic valve stenosis include the medical history and physical examination findings. The main clinical manifestations are angina pectoris, stroke, dyspnea on exertion and, ultimately, heart failure. In physical examinations, the arterial pulse may be found to be small and sustained, the cardiac pulse may be shifted inferiorly and laterally by left heart failure, and the systolic thrill may be palpable in the second intercostal space bilaterally. Finally, cardiac auscultation may reveal a single second sound and a systolic murmur with a late peak, heard more clearly at the base of the heart. All the physical examination findings, including delayed ascending carotid pulse, loud and prolonged systolic murmur and single second heart sound, have been found to correlate with stenosis.^5^

After the onset of these symptoms, the mean survival is 4.5 years with angina, 2.6 years with dizziness on exertion and one year with the presence of left heart failure limitation. These symptoms occur primarily through low cardiac output and decreased coronary flow. The survival in the advanced stage of the disease is short: 20% over a three-year period for patients with major left ventricle impairment in functional class III or IV of the New York Heart Association (NYHA). The mortality rate is non-linear and approximately 10% of patients die during the first six months after the onset of symptoms.^4^

To evaluate disease progression, all patients should be tested using Doppler echocardiography, which is the gold standard test for diagnosing the disease. Patients with moderate aortic stenosis should undergo this test once a year and those with severe symptoms that are close to indications for surgery should undergo Doppler echocar-diography every six months.^4^ A transthoracic echocardiography is useful for detecting valvular calcifications, designing leaflets, determining the severity of stenosis (through viewing the hole) and enabling calculations on the pressure gradient that is formed between the left ventricle and aorta.

The limit that is considered normal for aortic valve opening is 1.6 - 2.6 cm.^4^ The actual size of the aortic valve orifice is not usually visible by means otransthoracic echocardiography. Thus, transoesophageal echocardiography may also be useful for calculating direct measurements on the valve orifice in patients with aortic valve stenosis. However, from a practical standpoint, it is unnecessary to determine the valve area. The echocardiographic criterion for considering that the aortic valve stenosis is clinically significant is the presence of a gradient greater than 50 mmHg,^4,5^ which corresponds to a peak aortic jet velocity of 3.5 m/s.^6^

Stress tests may also be useful for apparently asymptomatic patients, in order to detect hidden symptoms, limited ability to exercise and abnormal blood pressure responses. Tension on exercising must be absolutely avoided for symptomatic patients.^5^ Such patients, with severe disease, are candidates for surgery, since clinical therapy has little to offer. However, clinical treatment may be necessary for patients who are considered inoperable. The drugs that may apply in these cases are digitalis, angiotensin-converting enzyme inhibitors and diuretics, and beta-blockers should be avoided because they decrease myocardial function.^5^

Currently, aortic valve stenosis is the main indication for valve replacement in North America and Europe. In the United States, 16,330 single procedures and 14,976 procedures associated with myocardial revascularization were reported by the Society of Thoracic Surgeons in 2006.^2^ Thus, cardiac surgery with extracorporeal circulation and aortic valve replacement with mechanical or biological prostheses has been the procedure of choice in severe cases. Conventional surgery provides symptomatic improvement and increased survival for most patients with low surgical risk.^7^ However, in patients with associated diseases, very elderly patients, cases of reoperation and cases of severe ventricular dysfunction, the mortality rate can reach 50%.^8,9^

Recently, the concept of degenerative valve disease has been replaced by evidence of an active inflammatory process that in many ways is related to atherosclerosis (elevation of LDL cholesterol and its oxidation by macrophages) and coronary artery disease.^10^ Therefore, the effect of therapy to reduce lipid levels in relation to progression of aortic valve calcification has begun to be tested. Several prospective studies have suggested that statins may provide benefits for these patients.^11,12^

In 1976, the Japanese scientist Akira Endo identified a fungal metabolite that blocks cholesterol synthesis by inhibiting the enzyme 3-hydroxy-3-methylglutaryl coenzyme A (HMG-CoA) reductase. This resulted in the first “statin” agent, mevastatin.^13^ Statins are substances capable of decreasing the intracellular synthesis of cholesterol by competing with HMG-CoA reductase, thereby partially inhibiting its action. This partial inhibition also ensures formation of the cholesterol necessary for balancing the cell membrane and synthesizing steroid hormones and vitamin D.^14^

Absorption and excretion of statins occur in the gut, and their metabolization occurs in the liver. All statins are metabolized to a greater or lesser degree in cytochrome P 450, through different enzyme systems. In addition to reducing intracellular cholesterol synthesis, statins increase LDL receptors, particularly in the liver, thus allowing greater clearance of these particles and consequently decreasing their plasma levels.^14^

Additionally, statins interfere with the secretion of very low density lipoproteins (VLDL), which contributes towards reducing the LDL in circulation. Lower production of VLDL and higher capture of VLDL remnants are responsible for a decrease in triglycerides. Statins slightly reduce the levels of high-density lipoprotein (HDL), probably by decreasing the activity of protein-cholesterol ester transfer and increasing the synthesis of apolipoprotein Al.^14^

In practice, at the usual doses, statins reduce LDL levels by 7% and triglycerides by 22-45%, and increase HDL levels by 5-10%. These changes differ for each drug and are dose-dependent. Doses should be checked after 15 days of use, and they stabilize after one month of treatment.^14^

In addition to blocking this key enzyme involved in cholesterol synthesis, statins have several pleiotropic properties, including: increased nitric oxide in cases of endothelial dysfunction, upregulation of the expression of endothelin-1, antioxidant effects, anti-inflammatory properties, stabilization of atherosclerotic plaques, anticoagulant effects and inhibition of graft rejection after kidney and heart transplantation.^13^

Although the role of the pleiotropic effects of statins in cardiovascular prevention remains to be determined, statins have became one of the classes of drugs most commonly sold up to the present day, since their introduction to the market in 1986. Currently, the drugs available commercially in Brazil and the United States, in order of release are: lovastatin, pravastatin, fluvastatin, atorvastatin, simvastatin and rosuvastatin.^13,15^

Since the introduction of statins, many clinical trials have demonstrated the importance of statins in primary and secondary prevention of cardiovascular disease, especially coronary artery disease. The Myocardial Ischaemia Reduction with Aggressive Cholesterol Lowering Study (MIRACL Trial), for example, was a randomized, double-blind, controlled trial comparing atorvastatin with placebo among patients with an acute coronary syndrome event (unstable angina or acute myocardial infarction without ST elevation).^16^

The primary endpoint of the MIRACL Trial^16^ was to determine whether treatment with atorvastatin at a dose of 80 mg/day, starting within the first 24 to 96 hours would reduce mortality, nonfatal ischemic events, heart failure, recurrent symptoms of myocardial ischemia requiring hospitalization, and calcification. These outcomes occurred in 228 patients (14.8%) in the atorvastatin group and 269 patients (17.4%) in the placebo group (relative risk, RR = 0.84; 95% confidence interval, CI: 0.70 to 1.0; P = 0.048).

The MIRACL Trial^16^ found that there were no significant differences in the risks of death, non-fatal acute myocardial infarction or cardiac arrest between the atorvastatin group and the placebo group, although the atorvastatin group had a lower risk of symptoms of recurrent ischemia with rehospitalization (6.2% versus 8.4%; RR = 0.74; 95% CI: 0.57 to 0.95; P = 0.02). There was also no significant difference between the atorvastatin group and the placebo group with regard to secondary outcomes, including surgical or percutaneous myocardial revascularization, nonfatal stroke, heart failure and angina with rehospitalization, such that there were no signs of myocardial ischemia or changes in lipid profile between the beginning and the end of the follow-up (RR = 1.01; 95% CI: 0.88 to 1.15; for any event). In the atorvastatin group, the plasma levels of LDL cholesterol decreased from 124 mg/dl to 72 mg/dl. Abnormal liver transaminase levels (three times above normal) were found more frequently in the atorvastatin group than in the placebo group (2.5% versus 0.6%, P = 0.001). The conclusion from this study was that for patients with acute coronary syndrome, lipid-lowering therapy with atorvastatin at a dose of 80 mg/day reduced recurrent ischemic events during the first 16 weeks, especially the symptoms of recurrent ischemia with rehospitalization.^16^

Another very important study on primary prevention of cardiovascular events, with an impact on the world scientific community, was the Rosuvastatin to Prevent Vascular Events in Men and Women with Elevated C-Reactive Protein (Jupiter Trial).^17^ Since statins decrease the levels of high-sensitivity C-reactive protein (CRP) as well as cholesterol levels, this trial hypothesized that people with elevated high-sensitivity CRP levels but without hyperlipidemia might benefit from statin treatment, because elevated CRP levels predispose towards cardiovascular events.

This randomized double-blind controlled trial^17^ selected 17,802 healthy men and women with LDL cholesterol levels of less than 130 mg/dl (3.4 mmol/l) and high-sensitivity CRP levels of 2.0 mg/l or higher, who were given rosuvastatin 20 mg/day or placebo. The subjects were followed up to monitor for occurrences of the combined primary outcomes of myocardial infarction, stroke, arterial revascularization, hospitalization due to unstable angina or death from cardiovascular causes. The trial was stopped after a mean follow-up of 1.9 years (maximum, 5.0). Rosuvastatin reduced LDL cholesterol levels by 50% and high-sensitivity CRP levels by 37%.

The primary outcome rates were 0.77 and 1.36 per 100 person-years of follow-up in the rosuvastatin and placebo groups, respectively (hazard ratio [HR] for rosuvastatin, 0.56; 95% CI, 0.46 to 0.69; P < 0.00001), with corresponding rates of 0.17 and 0.37 for myocardial infarction (HR, 0.46; 95% CI, 0.30 to 0.70; P = 0.0002); 0.18 and 0.34 for stroke (HR, 0.52; 95% CI, 0.34 to 0.79; P = 0.002); 0.41 and 0.77 for revascularization or unstable angina (HR, 0.53; 95% CI, 0.40 to 0.70; P < 0.00001); 0.45 and 0.85 for the combined outcome of myocardial infarction, stroke or death from cardiovascular causes (HR, 0.53; 95% CI, 0.40 to 0.69; P < 0.00001); and 1.00 and 1.25 for death from any cause (HR, 0.80; 95% CI, 0.67 to 0.97; P = 0.02). Thus, in this trial on apparently healthy individuals without hyperlipidemia but with elevated high-sensitivity CRP levels, rosuvastatin significantly reduced the incidence of major cardiovascular events.^17^

Given the clinical association with hypercholesterolemia and coronary artery disease, and the histological similarities with atheroma, it has been suggested that statin therapy may halt the progression, or even induce regression of calcific aortic valve stenosis, through its mechanisms of action. Several retrospective observational studies conducted a few years ago showed that statin therapy was associated with delayed disease progression and showed that the rate of change in aortic jet velocity went down by 0.30 m per second per year and that rate of change in valvular calcification went down by 30%.^18-23^

Recently, new studies have appeared, with the same proposition of examining the relationship between statins and the progression of aortic valve stenosis. The TASS trial (Tyrolean Aortic Stenosis Study), for example, had a prospective, randomized, placebo-controlled design and characterized the natural history, risk factors and possible modulation of calcified aortic valve stenosis through administration of atorvastatin 20 mg/day, versus placebo. However, this study did not show that treatment with atorvastatin was effective in relation to progression of calcific aortic stenosis through improving the pressure gradient between the aortic valve and left ventricle (RR = 1.02; 95% CI: 0.96 to 1.08; P = 0551).^24^

On the other hand, in the non-randomized, prospective, observational and open RAAVE study (Rosuvastatin in Endothelial Dysfunction in Aortic Valve), a change in the size of the aortic valve in the control group from -0.10 ± 0.09 cm^2^ per year was found, compared with -0.05 ± 0.12 cm^2^ per year in the rosuvastatin group (P = 0.041). Furthermore, the increase in peak velocity of the aortic valve was 0.24 ± 0.30 m/s/year in the control group, compared with 0.04 ± 0.38 m/s/year in the rosuvastatin group (P = 0.007). These data indicate that within this hypothesis, slowing of the progression of aortic disease was found through echocardiography.^25^

Given that there is no robust scientific evidence to change clinical practice, patients with aortic valve stenosis are still being subjected to invasive procedures such as exchange of the valve apparatus. Patients often remain asymptomatic for long periods, while the disease becomes established through a constant inflammatory process.

Within this scenario, when diagnosed, patients are already at an advanced stage of the disease process, with significant valve narrowing, deterioration of myocardial function and poor quality of life. Moreover, many patients are also exposed to chronic diseases such as diabetes mellitus, hypertension, dyslipidemia and smoking, which hinder the postoperative recovery if they have to undergo a surgical procedure. They may have to spend a long time in hospital, with burdensome costs for the health service.

Therefore, to allow new decisions to be made regarding clinical interventions such as the use of statins for treating aortic valve stenosis, it is important that evidence should be available from a systematic review of randomized controlled trials using the Cochrane methodology. On this topic, no such review currently exists. Systematic reviews of the literature are studies that integrate the results from primary studies and provide a basis for rational decision-making.^26^

Systematic reviews make it possible to distinguish between effective and ineffective types of treatment, clarify controversies about treatments, determine the clinical decisions that need to be implemented, and limit the chances of systematic and random errors in the analysis, thereby providing results with greater reliability.^27-29^

## CONCLUSION

We believe that this review is important because of the impact that aortic valve stenosis has had in the Western world, especially among the elderly. Thus, a systematic review of all randomized controlled trials identified and selected from the search strategy that address the safety and effectiveness of treatment with hydroxy-methylglutaryl-CoA reductase (statins) for patients with aortic valve stenosis is currently in progress at the Brazilian Cochrane Centre in conjunction with the Heart Group of the Cochrane Collaboration based at the Department of Non-Communicable Disease Epidemiology, London School of Hygiene & Tropical Medicine, United Kingdom.
